# Modeling and Analysis of a Composite Structure-Based Soft Pneumatic Actuators for Soft-Robotic Gripper

**DOI:** 10.3390/s22134851

**Published:** 2022-06-27

**Authors:** Ming Yu, Wenwen Liu, Jian Zhao, Yanyan Hou, Xuewu Hong, Hongjie Zhang

**Affiliations:** 1 School of Computer and Information Engineering, Tianjin Chengjian University, Tianjin 300384, China; 2 Tianjin Key Laboratory of Modern Mechatronics Equipment Technology, School of Mechanical Engineering, Tiangong University, Tianjin 300387, China; 2031050606@tiangong.edu.cn; 3 School of Control and Mechanical Engineering, Tianjin Chengjian University, Tianjin 300384, China; zhaojian@tcu.edu.cn (J.Z.); houyanyan@tcu.edu.cn (Y.H.); hong_xw@tcu.edu.cn (X.H.)

**Keywords:** soft pneumatic actuator, analytical modeling, bending angle model, kinematic model, soft robotic system

## Abstract

Soft pneumatic actuators are extensively used in soft robots, and their bending angles and kinematic rules at different pressures play a crucial role in practical applications. This investigation aims to model the bending angle and motion of a new type of soft pneumatic actuator that adopts a composite structure consisting of two kinds of pneumatic networks. Based on the structural and deformation characteristics of the proposed soft actuator, the constitutive model is established, and then the moment equilibrium and virtual work principle are combined to model the bending angle of two pneumatic modules. The kinematic model of the proposed soft actuator is co-opted from the kinematic modeling of rigid robots. By employing the piecewise constant curvature method and coordinate transformation, the location of any chamber of the soft actuator can be calculated. The effectiveness of the developed analytical models is then tested, and the calculated results show good agreement with the experimental results. Finally, three soft actuators are used to constitute a soft gripper, and the pinching and enveloping grasping performance are examined. All experimental test results demonstrate that the developed bending angle and kinematic models can explain the bending principle of the proposed soft actuators well.

## 1. Introduction

Characterized by light weight, excellent compliance, strong adaptability, easy control, and high security, soft robots have attracted wide interest in recent years and present growing applications in grasping [1,2], rehabilitation [3,4], locomotion [5,6], and manipulation [7,8]. As one of the most important components of the soft robotic system, the soft actuator has a significant influence on the performance of soft robots. At present, the soft pneumatic actuator (SPA) is a common actuation method that has been widely used, involving pneumatic network actuators (PneuNets) [9,10], fiber-reinforced actuators [11,12], and pneumatic artificial muscles (PAMs) [13,14]. Compared with the latter two SPAs, the PneuNets have distinct characteristics, such as ease of fabrication and bidirectional bending ability, and have been receiving growing attention from the SPA community. However, since the PneuNets are usually made of soft and elastic material, they do not have enough pressure-bearing capacity and rigidity, and as a result, their small output force is often criticized.

The mechanical and kinematic properties of soft PneuNets can be understood by numerical and analytical modeling. As one of the most important tools within the design and performance investigation of PneuNets, finite element analysis (FEA) technology is widely used [15,16]. Although commercial FEA software can help us to analyze the mechanical and kinematic properties of PneuNets, the development of the FEA model is often a time-consuming process. In the meanwhile, it is not easy to figure out the influence that the model parameters have on the performance of the PneuNets. Moreover, the FEA-based analysis method usually takes up significant computing resources. Compared with the FEA method, analytical modeling involves a systematic application of basic physical laws, and it can describe the relationships among the structural parameters, applied pressure, and the mechanical and kinematic properties of the PneuNets via equations; therefore, it has simpler and more intuitive expression, and it is also easier to understand and use in practical application. However, the complexity of the chamber structure, the nonlinear relationship between the input pressure and motion, and the hysteresis phenomenon within the inflating and deflating process bring difficulty to the establishment of an accurate analytical model. 

To develop the analytical model to describe the bending angle rules of PneuNets with changes in the input pressure, there are several main methods. One method aims to calculate the deformation displacement of the contact points between the neighboring chambers of the PneuNets first using rigid body theory. Depending on the structural parameters and geometric relationship, a calculation model for the bending angle can be developed [17]. Another method regards the chamber of the PneuNets as a cantilever beam. Based on the bending moment calculation, the quasi-static bending displacement can be evaluated [18,19,20]. The above two kinds of methods ignore the large-strain and nonlinear characteristics of the elastic material, as well as the bending principle of the PneuNets; hence, they have poor adaptability to different PneuNets. To describe the deformation characteristics of PneuNets more accurately, strain energy density functions are used to reflect the mechanical properties of the soft elastic material; examples of this include the Neo-Hookean model [21,22], Yeoh model [23,24], and Mooney–Rivlin model [25,26]. During the modeling, the hyperelastic material is assumed to be incompressible and to bend with constant curvature. By developing the constitutive model, the relationship between stress and strain of the chamber can be achieved. According to the moment equilibrium or virtual work principle, the bending angle model of the PneuNets can be constructed. Kinematic modeling is also a fundamental issue in the application of soft PneuNets. At present, kinematic modeling of the soft actuator mainly depends on the rigid theory, in which the actuator is viewed as the connection of a series of cantilever beams. Therefore, the kinematic modeling of the traditional rigid robotic system can be co-opted [27,28]. In the practical modeling process, the piecewise constant curvature (PPC) method and the transformation of coordinates are extensively used [29,30,31].

As noted earlier, the output force of traditional PneuNets is relatively small. When PneuNets are applied to grasping, the insufficient output force can cause instability and unreliability of the grasping. To improve the performance of the traditional soft actuator, some composite-structure-based soft actuators were attempted. For example, Ref. [32] designed a novel rigid-flexible soft actuator, which realized the bending action of a human-like finger and improved the bearing capacity of the soft actuator, presenting good engineering value. In our recent research, we proposed a new type of SPA that adopts a composite structure consisting of two kinds of pneumatic networks. Compared with the traditional single-pneumatic-network-based soft actuator, the proposed one combines the advantages of the two typical pneumatic network structures. Performance test results prove that the proposed soft actuator significantly enhances the output force and keeps an expected deformation ability in the meanwhile. To promote the practical application of the proposed soft actuator, this investigation aims to develop its bending angle model and kinematic model. For this purpose, the structural characteristics and motion principle of the proposed soft actuator were analyzed, and the Yeoh constitutive model, moment equilibrium [33], virtual work principle [34], PPC method, and coordinate transformation were combined to finish the analytical modeling. The performance test results indicate that the analytical models have good agreement with the experimental results. Finally, a soft gripper is assembled using three soft actuators. Based on the developed kinematic model, grasping experiments are carried out, and the results further demonstrate that the bending angle and kinematic model can explain the deformation and motion principle of the proposed soft actuator well, laying a good foundation for control of the actuator in practical applications.

## 2. Structural Characteristics and Bending Principle of New PneuNets

**Figure 1:**
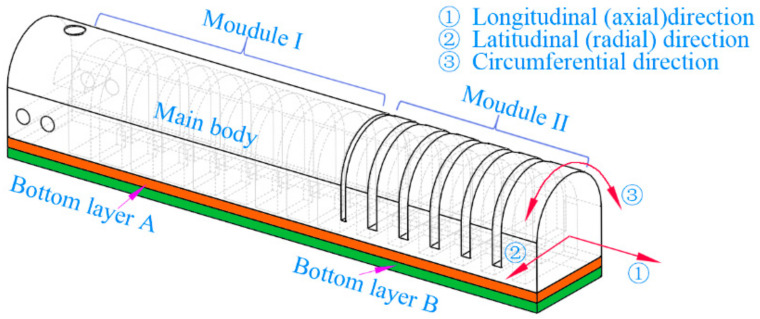
Structural schematic diagram of the proposed SPA.

The structure of the newly proposed soft PneuNets is illustrated schematically in Figure 1. The soft PneuNet consists of two types of pneumatic networks, involving a slow pneumatic network (SPN) at its root and a fast pneumatic network (FPN) at the tip. Via a soft joint and two bottom layers with an inextensible paper inserted in between, two soft pneumatic modules are connected to constitute a composite structure.

**Figure 2:**
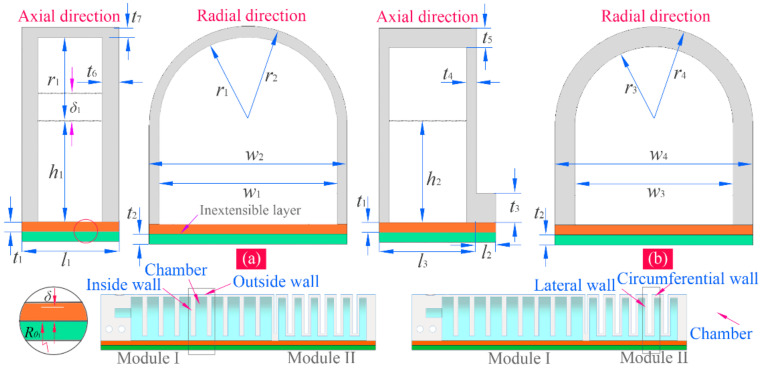
Cross-sections of the soft chambers: (**a**) chamber of module one and (**b**) chamber of module two.

Module one of the soft actuator has nine chambers, and module two has six chambers. Cross-sections of two kinds of chambers along the axial direction and radial direction are shown in Figure 2, where the primary geometric parameters are labeled; their relevant values are listed in Table 1. When inflated, the chamber of module one will expand and deform mainly along the axial direction of the soft actuator since the thickness of its outside wall is smaller than those of the inside walls between the adjacent chambers. In the meanwhile, the pressure on both sides of each inside wall is equal. Compared with the bending deformation of the chamber of module one, that of module two presents a different form. Because the thickness of the lateral wall is smaller relative to that of other walls, the lateral wall is easier to expand, making the expanded lateral walls of the adjacent chambers squeeze each other. Owing to the existence of the inextensible layers, two modules will bend to the side of the inextensible layers.

Due to having different structural characteristics, two pneumatic modules of the proposed soft actuator have different output properties. Compared with module two, module one can withstand a larger pressure; as a result, it can output a bigger force, although its deformability gets worse. Module two possesses better deformability, allowing it to generate a bigger bending angle; however, its output force is relatively small. The proposed soft PneuNet combines the advantages of the two modules. When used to constitute a soft-robotic gripper, module one can improve the output force of the whole SPA, and module two is conducive to forming a larger contact area due to its excellent formability, enabling the gripper to be competent for grasping larger, heavier, and irregular objects and giving it strong adaptability and stability whether in pinching or in enveloping grasping. To figure out the changing rules of the bending angle and motion of the proposed SPA versus the pressure, the following investigations are carried out.

## 3. Modeling of the Proposed SPA

**Figure 3:**
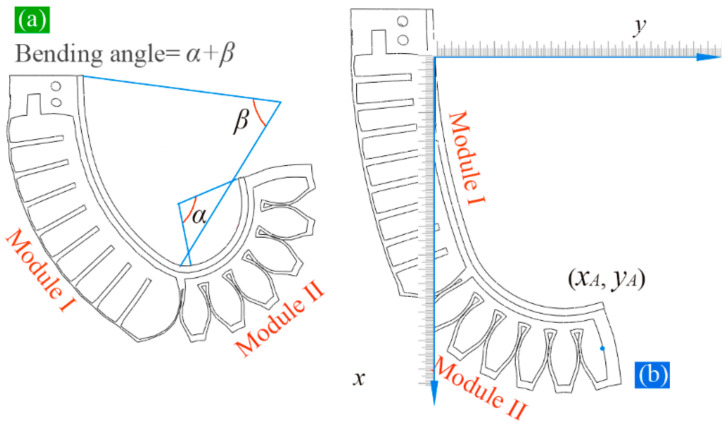
(**a**) Definition of the bending angles and (**b**) a schematic diagram for the measuring of the motion range.

It is well known that the bending deformation and motion characteristics of the SPA are very important. This part introduces the analytical models of the proposed SPA in detail. Section 3.1 provides an introduction to the constitutive model of the Yeoh hyperelastic model. Based on the constitutive model, Section 3.2 establishes the analytical model of the total bending angle of the proposed soft PneuNets. Before modeling, the bending angles of the soft actuator are defined, as shown in Figure 3a, where β, α, and β+α denote the bending angles of module one, module two, and whole actuator, respectively. In Section 3.3, a kinematical model of the actuator is built up, based on which the location that any chamber can reach can be determined, as schematically shown in Figure 3b.

### 3.1. Constitutive Model

The elastic behavior of the material of the chamber wall is described with an incompressible Yeoh hyperelastic model. In hyperelasticity, the specific relationship between the in-plane stress (tension) and strain (stretch) depends on the strain energy density function $W=W\left(I_{1},I_{2},I_{3}\right)$, where $I_{1}$, $I_{2}$, and $I_{3}$ are three invariants of the Cauchy–Green tensor, and they are given by the following:

(1) $$\left\{\begin{array}{l} I_{1}=\lambda_{1}{}^{2}+\lambda_{2}{}^{2}+\lambda_{3}{}^{2}\\ I_{2}=\lambda_{1}{}^{2}\lambda_{2}{}^{2}+\lambda_{2}{}^{2}\lambda_{3}{}^{2}+\lambda_{1}{}^{2}\lambda_{3}{}^{2}\\ I_{3}=\lambda_{1}{}^{2}\lambda_{2}{}^{2}\lambda_{3}{}^{2} \end{array}\right.$$

where $\lambda_{1}$, $\lambda_{2}$, and $\lambda_{3}$ denote the axial, radial, and circumference stretch. Note that the material is assumed to be incompressible; therefore, the third invariant $I_{3}$ is equal to one, and the first two invariants can be written as:

(2) $$\left\{\begin{array}{c} I_{1}=\lambda_{1}{}^{2}+\lambda_{2}{}^{2}+\lambda_{3}{}^{2}\\ I_{2}=\frac{1}{\lambda_{1}{}^{2}}+\frac{1}{\lambda_{2}{}^{2}}+\frac{1}{\lambda_{3}{}^{2}} \end{array}\right.$$

According to the Yeoh model, the strain energy density function can be written as:

(3) $$W={\sum_{i=1}^{N}C_{i0}\left(I_{1}-3\right)}^{i}+\sum_{k=1}^{N}\frac{1}{d_{k}}{\left(J-1\right)}^{2k}$$

where N denotes the order of the strain energy density function, and $d_{k}$ and $C_{i0}$ are two material constants. For the incompressible material, J is equal to one, and thus, when N=2, Equation (3) can be expressed as:

(4) $$W=C_{10}\left(I_{1}-3\right)+C_{20}{\left(I_{1}-3\right)}^{2}$$

As mentioned above, when the chamber is inflated, its expansion and deformation are mainly along the axial (longitudinal) direction of the soft actuator. If assuming there is no deformation in the circumference direction, we can get $\lambda_{3}=1$. Set $\lambda_{1}=\lambda$ (the axial stretch); then, $\lambda_{2}=1/\lambda$ (the radial stretch), and thus $I_{1}=I_{2}=\lambda^{2}+1/\lambda^{2}+1$. Equation (4) can then be changed to:

(5) $$W=C_{10}{\left(\lambda -\frac{1}{\lambda }\right)}^{2}+C_{20}{\left(\lambda -\frac{1}{\lambda }\right)}^{4}$$

By calculating the derivative of the strain energy density with respect to stretch, we can obtain the relationship between stress and strain:

(6) $$\sigma_{i}=\frac{\partial W}{\partial \lambda_{i}}=\frac{\partial W}{\partial I_{1}}\frac{\partial I_{1}}{\partial \lambda_{i}}+\frac{\partial W}{\partial I_{2}}\frac{\partial I_{2}}{\partial \lambda_{i}}+\frac{\partial W}{\partial I_{3}}\frac{\partial I_{3}}{\partial \lambda_{i}}$$

### 3.2. Bending Angle of the Soft Actuator

The proposed actuator is made of Dragon Skin 30 silicone rubber, which is widely used in soft PneuNets. According to the relevant literature, the material constants $C_{10}$ and $C_{20}$ are equal to 0.11 and 0.02, respectively, when the Yeoh hyperelastic model is used [35,36,37]. Based on Equations (5) and (6), the following equation can be obtained:

(7) $$\sigma =\frac{\partial W}{\partial \lambda }=2\left(\lambda -\frac{1}{\lambda^{3}}\right)\left[C_{10}+2C_{20}{\left(\lambda -\frac{1}{\lambda }\right)}^{2}\right]$$

When ignoring the quadratic term and up, the above equation can be reduced to:

(8) $$\sigma =8C_{10}\left(\lambda -1\right)$$

**Figure 4:**
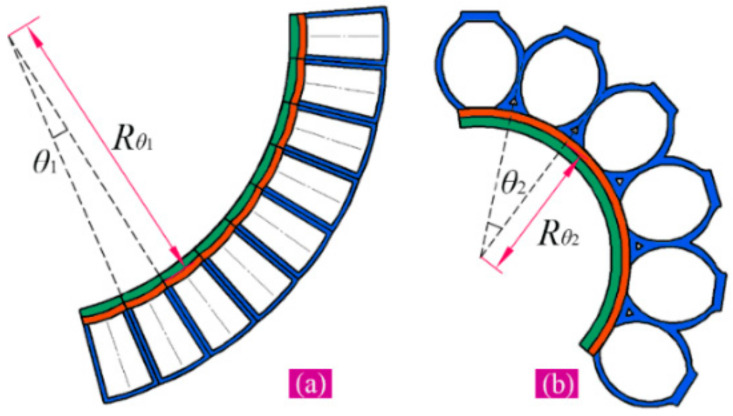
Bending deformation of the two modules: (**a**) module one and (**b**) module two.

Figure 4 describes the bending deformation of two pneumatic modules. When inflated, every chamber of module one can generate a bending angle of $\theta_{1}$ (Figure 4a), and that of module two can give a bending angle of $\theta_{2}$ (Figure 4b). According to the relationship between arc length and bending angle, we can calculate the longitudinal stretch of a certain position that has a distance of ±δ to the faying surface of two inextensible layers (see the inset of Figure 2a):

(9) $$\lambda_{\delta }=\left(R_{\theta_{1}}\pm \delta \right)\theta_{1}/l_{1}$$

where $l_{1}$ denotes the original length of the chamber without inflation, and $R_{\theta_{1}}$ corresponds to the bending radius of the inextensible layer. Combine Equations (8) and (9), and the axial stress of any layer within the chamber caused by the bending deformation can be calculated. For example, the longitudinal stress of a certain layer that is $\delta_{1}$ distant from the layer $h_{1}$ (see Figure 2a) can be written as:

(10) $$\sigma \left(\lambda_{\delta_{1}}\right)=8C_{10}\left(\frac{\left(R_{\theta_{1}}+t_{1}+h_{1}+\delta_{1}\right)\theta_{1}}{l_{1}}-1\right)$$

The driving moment of the bending deformation within the chamber of module one can be given by:

(11) $$M_{D}=\int_{0}^{h_{1}}P_{1}W\left(\delta +t_{1}\right)d\delta +2\int_{0}^{r_{1}}P_{1}\sqrt{r_{1}{}^{2}-\delta^{2}}\left(\delta +h_{1}+t_{1}\right)d\delta$$

where $P_{1}$ denotes the applied pressure. The resisting moment generated by the silicone rubber can be written as:

(12) $$\begin{array}{l} M_{R}=\int_{0}^{t_{1}}\sigma \left(\lambda_{-t_{1}}\right)W\delta d\delta +\int_{0}^{t_{1}}\sigma \left(\lambda_{t_{1}}\right)\delta d\delta +2\int_{0}^{h_{1}}\sigma \left(\lambda_{h_{1}}\right)\left(t_{1}+\delta \right)d\delta \\ \;\;\;\;\;\;\;\;\;\;\;\;\;\;\;\;\;\;\;\;\;\;\;\;\;\;\;+2\int_{0}^{r_{1}}\sigma \left(\lambda_{r_{1}}\right)\left(\sqrt{r_{2}{}^{2}-\delta^{2}}-\sqrt{r_{1}{}^{2}-\delta^{2}}\right)\left(t_{1}+h_{1}+\delta \right)d\delta \\ \;\;\;\;\;\;\;\;\;\;\;\;\;\;\;\;\;\;\;\;\;\;\;\;\;\;\;+2\int_{0}^{t_{7}}\sigma \left(\lambda_{t_{7}}\right)\left(\sqrt{r_{2}{}^{2}-{\left(r_{1}+\delta \right)}^{2}}\right)\left(t_{1}+h_{1}+r_{1}+\delta \right)d\delta \end{array}$$

where $\sigma (\lambda_{-t_{1}})$, $\sigma (\lambda_{t_{1}})$, $\sigma (\lambda_{h_{1}})$, $\sigma (\lambda_{r_{1}})$, and $\sigma (\lambda_{t_{5}})$ are the longitudinal stress corresponding to different layers, and they can be obtained by referring to Equation (10). Thus, according to the moment equilibrium principle ($M_{D}=M_{R}$), we can develop the relationship model between the bending angle and the applied pressure of the chamber. Thus, the total bending angle of module one can be calculated by $n_{1}\theta_{1}\left(P_{1}\right)$, where $n_{1}$ denotes the chamber number.

The bending deformation of the chamber of module two is mainly caused by the mutual squeezing of the neighboring chambers, as shown in Figure 4b, which will result in the variation of some geometric parameters; therefore, the analytical model will be developed based on the principle of virtual work [38]. Assuming that there is no external force acting on the chamber, the work of the internal pressure can be equal to the stored energy associated with the deformation of the chamber, namely,

(13) $$P_{2}\cdot dV_{p}=V_{s}\cdot dW$$

where $P_{2}$ denotes the applied pressure, and W is the strain energy density function. $V_{P}$ and $V_{S}$ are the volumes of the air cavity within the chamber and the basal body of the silicone rubber (walls), respectively. $V_{S}$ can be calculated using the dimension parameters listed in Figure 2b,

(14) $$V_{S}=l_{3}\left[\frac{\left(\pi r_{4}{}^{2}\right)}{2}+w_{4}\left(h_{3}+2t_{1}\right)\right]+(2t_{1}+t_{3})w_{4}l_{2}-\left(l_{3}-2t_{4}\right)\left[\frac{\left(\pi r_{3}{}^{2}\right)}{2}+w_{3}h_{2}\right]$$

The total volume of an expanded chamber can be calculated approximatively through

(15) $$V_{T}=\lambda_{\theta_{2}}\left[l_{3}\frac{\left(\pi r_{4}{}^{2}\right)}{2}+l_{3}w_{4}\left(h_{2}+2t_{1}\right)+(2t_{1}+t_{3})w_{4}l_{2}\right]$$

where $\lambda_{\theta_{2}}$ is the longitudinal stretch of a single chamber of module two. According to Figure 4b, it can be calculated by

(16) $$\lambda_{\theta_{2}}=\frac{R_{\theta_{2}}\theta_{2}}{R_{\theta_{2}}\sin\theta_{2}}=\frac{\theta_{2}}{\sin\theta_{2}}$$

Thus, $V_{p}=V_{T}-V_{S}$. We differentiate $V_{p}$ and W in Equation (13) with respect to $\theta_{2}$ and achieve

(17) $$\frac{dV_{p}}{d\theta_{2}}=\frac{d\lambda_{\theta_{2}}}{d\theta_{2}}\left[l_{3}\frac{\left(\pi r_{4}4^{2}\right)}{2}+l_{3}w_{4}\left(h_{2}+2t_{1}\right)+(2t_{1}+t_{3})w_{4}l_{2}\right]$$

(18) $$\frac{dW}{d\theta_{2}}=2\lambda_{\theta_{2}}\frac{d\lambda_{\theta_{2}}}{d\theta_{2}}\left(\left.1-\frac{1}{\lambda_{\theta_{2}}{}^{4}}\right)\right.\left[C_{10}+2C_{20}{\left(\lambda_{\theta_{2}}-\frac{1}{\lambda_{\theta_{2}}}\right)}^{2}\right]$$

where

(19) $$\frac{d\lambda_{\theta_{2}}}{d\theta_{2}}=\frac{\sin\theta_{2}-\theta_{2}\cos\theta_{2}}{{\left(\sin\theta_{2}\right)}^{2}}$$

Combining Equations (17)–(19), we can establish the relation model among the bending angle of a single chamber, applied pressure, and geometric parameters

(20) $$P_{2}\left(\theta_{2}\right)=\frac{2\frac{\theta_{2}}{\sin\theta_{2}}\left(\frac{\sin\theta_{2}-\theta_{2}\cos\theta_{2}}{{\left(\sin\theta_{2}\right)}^{2}}\right)\left(1-\frac{1}{{\left(\theta_{2}/\sin\theta_{2}\right)}^{4}}\right)\left[C_{10}+2C_{20}{\left(\frac{\theta_{2}}{\sin\theta_{2}}-\frac{\sin\theta_{2}}{\theta_{2}}\right)}^{2}\right]V_{S}}{\left(\frac{\sin\theta_{2}-\theta_{2}\cos\theta_{2}}{{\left(\sin\theta_{2}\right)}^{2}}\right)\left[l_{3}\frac{\pi r_{4}{}^{2}}{2}+l_{3}w_{4}\left(h_{2}+2t_{1}\right)+(2t_{1}+t_{3})w_{4}l_{2}\right]}$$

So far, the total bending angle can be obtained according to the definition noted above, and it can be expressed as $\theta_{T}=n_{1}\theta_{1}\left(P_{1}\right)+n_{2}\theta_{2}\left(P_{2}\right)$, where $n_{2}$ is the chamber number of module two.

### 3.3. Kinematical Modeling

**Figure 5:**
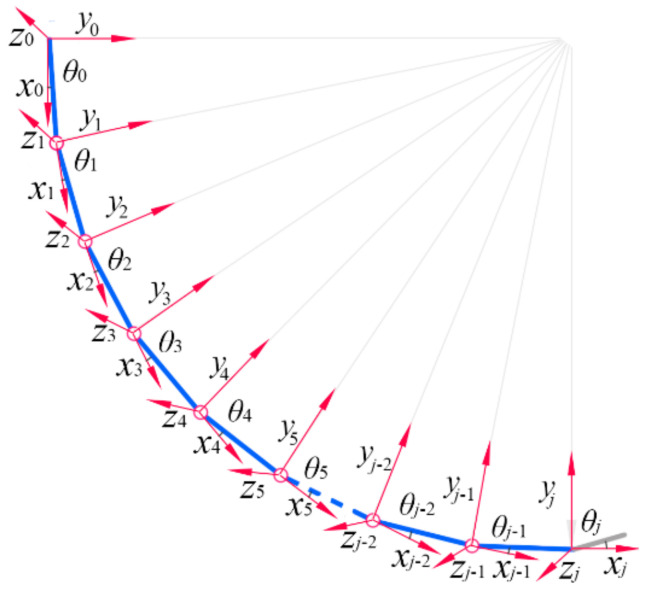
Simplification of the pneumatic module of the proposed SPA.

To develop the kinematical model of the proposed soft actuator, the piecewise constant curvature (PPC) method is co-opted. Since the chambers of each module have the same structure and dimension parameters, they will show identical deformation law and output properties. As we utilize the PPC method to construct the kinematical model, the inflated chambers from the same module are viewed as a series of arcs connected in series, and these arcs have the same curvature. Since each arc can correspond to one chord (or a cantilever beam), either pneumatic module of the proposed SPA can be simplified as shown in Figure 5, where each connecting joint between the adjacent chambers has been arranged with a local coordinate system: $O_{0}$ corresponds to the first chamber at the root of the soft actuator, $O_{1}$ corresponds to the joint connecting the first and second chambers, and so on. For the convenience of expression, $O_{0}$ is called the root joint. In each local coordinate system, the *z*-axis represents a rotation axis, and the tangent line of the arc crossing the joint is defined as the *x*-axis, with a positive direction toward the tip chamber. According to the right-hand rule, the positive direction of the *y*-axis can be determined. If the local coordinate system at the root joint is regarded as a base coordinate system, we can get the coordinates of other joints in this base coordinate system via coordinate transformation, relying on which the motion rule of any chamber in the base coordinate system can be described.

**Figure 6:**
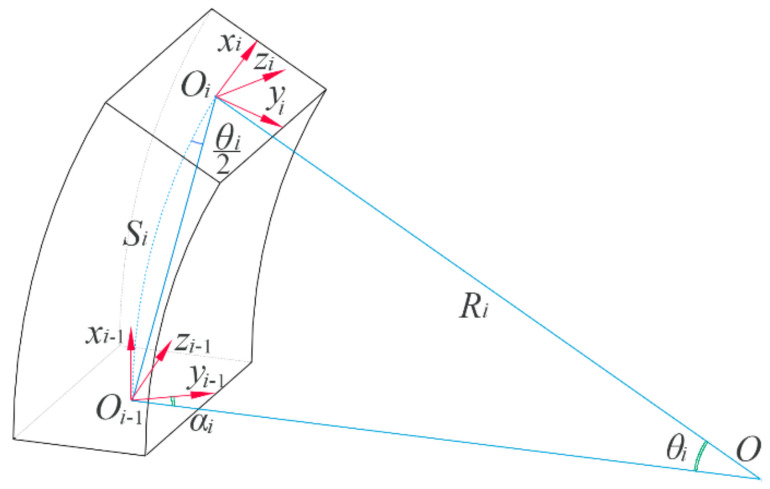
A schematic diagram for the coordinate transformation between two joints.

Based on the above analysis, it can be seen that the coordinate transformation between the root joint and other joints should be determined first. To do this, a small portion in the bottom layer B, which just corresponds to a complete chamber, is selected, as Figure 6 shows. At the connecting joints, two local coordinate systems, $O_{i-1}\left(x_{i-1},y_{i-1},z_{i-1}\right)$ and $O_{i}\left(x_{i},y_{i},z_{i}\right)$, are labeled. $\theta_{i}$ corresponds to the bending angle of this chamber, and $\alpha_{i}$ denotes a deflection angle between the positive directions of the $y_{i-1}$- axis and the plane formed by O, $O_{i-1}$, and $O_{i}$. It should be noted that the motion of the inflated soft actuator pertains to an in-plane motion; therefore, the deflection angle is equal to zero. To ensure universality, this deflection angle is retained. From Figure 6, the coordinate $O_{i-1}\left(x_{i-1},y_{i-1},z_{i-1}\right)$ can be seen to reach $O_{i}\left(x_{i},y_{i},z_{i}\right)$ through five steps. First, make $O_{i-1}$ rotate $-\alpha_{i}$ about the $x_{i-1}$-axis; then, revolve $\theta_{i}/2$ around the $z_{i-1}$-axis; third, move $l_{i}=2R_{i}\sin(\theta_{i}/2)$ along the positive direction of the new $x_{i-1}$-axis; fourth, rotate $\theta_{i}/2$ about the new $z_{i-1}$-axis; and finally, rotate $\alpha_{i}$ about the new $x_{i-1}$-axis. To achieve the homogeneous coordinate transformation matrix of each step, the Denavit–Hartenberg (DH) method is used. For the first step, its transformation matrix can be written as

(21) $$R_{x}\left(-\alpha_{i}\right)=\left[\begin{array}{cccc} 1 & 0 & 0 & 0\\ 0 & \cos\alpha_{i} & \sin\alpha_{i} & 0\\ 0 & -\sin\alpha_{i} & \cos\alpha_{i} & 0\\ 0 & 0 & 0 & 1 \end{array}\right]$$

For the second step, the transformation matrix can be written as

(22) $$R_{z}\left(\frac{\theta_{i}}{2}\right)=\left[\begin{array}{cccc} \cos\left(\frac{\theta_{i}}{2}\right) & -\sin\left(\frac{\theta_{i}}{2}\right) & 0 & 0\\ \sin\left(\frac{\theta_{i}}{2}\right) & \cos\left(\frac{\theta_{i}}{2}\right) & 0 & 0\\ 0 & 0 & 1 & 0\\ 0 & 0 & 0 & 1 \end{array}\right]$$

For the third step, the transformation matrix can be written as

(23) $$T_{x}\left(l_{i}\right)=\left[\begin{array}{cccc} 1 & 0 & 0 & 2R_{i}\sin\left(\frac{\theta_{i}}{2}\right)\\ 0 & 1 & 0 & 0\\ 0 & 0 & 1 & 0\\ 0 & 0 & 0 & 1 \end{array}\right]$$

For the fourth step, the transformation matrix can be written as

(24) $$R_{z}\left(\frac{\theta_{i}}{2}\right)=\left[\begin{array}{cccc} \cos\left(\frac{\theta_{i}}{2}\right) & -\sin\left(\frac{\theta_{i}}{2}\right) & 0 & 0\\ \sin\left(\frac{\theta_{i}}{2}\right) & \cos\left(\frac{\theta_{i}}{2}\right) & 0 & 0\\ 0 & 0 & 1 & 0\\ 0 & 0 & 0 & 1 \end{array}\right]$$

For the fifth step, the transformation matrix can be written as

(25) $$R_{x}\left(\alpha_{i}\right)=\left[\begin{array}{cccc} 1 & 0 & 0 & 0\\ 0 & \alpha_{i} & -\sin\alpha_{i} & 0\\ 0 & \sin\alpha_{i} & \cos\alpha_{i} & 0\\ 0 & 0 & 0 & 1 \end{array}\right]$$

Thus, the whole transformation matrix can be written as

(26) $$T_{i-1}^{i}=R_{x}\left(-\alpha_{i}\right)R_{z}\left(\theta_{i}/2\right)T_{x}\left(l_{i}\right)R_{z}\left(\theta_{i}/2\right)R_{x}\left(\alpha_{i}\right)$$

In practical use, since there is no deflection angle $\alpha_{i}$, the above transformation matrix can be reduced to

(27) $$T_{i-1}^{i}=R_{z}\left(\theta_{i}/2\right)T_{x}\left(l_{i}\right)R_{z}\left(\theta_{i}/2\right)$$

For the proposed soft actuator, there are nine chambers in module one and six chambers in module two. Due to having different output properties, two modules of the proposed soft actuator can be thought of as two groups of “link mechanisms” connecting in series. Based on the bending angle models of the two types of chamber units, we can get the location of the actuator’s tip in the base coordinate system, and it can be given by

(28) $$T=\prod_{i=1}^{9}R_{z}\left(\theta_{1}/2\right)T_{x}\left(l_{1}\right)R_{z}{\left(\theta_{1}/2\right)}_{i}\prod_{i=10}^{15}R_{z}\left(\theta_{2}/2\right)T_{x}\left(l_{3}\right)R_{z}{\left(\theta_{2}/2\right)}_{i}$$

Based on Equation (28), the locations of any joints in the base coordinate system can be obtained as well.

### 3.4. Kinematical Model of a Soft Gripper Constituted by the Proposed Soft Actuators

**Figure 7:**
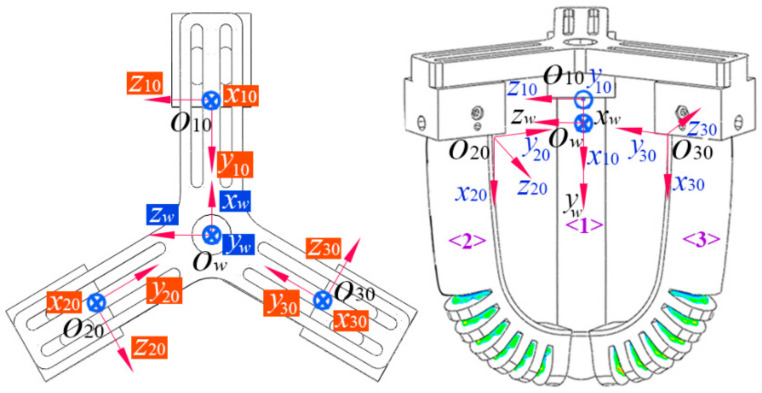
A three-finger soft gripper constituted by the proposed soft actuators and its coordinate system definition.

When applied to grasping, three soft actuators are installed on a Y-shape connector, constituting a three-finger soft gripper, as shown in Figure 7. To describe the kinematical rule of the soft actuators in the space enclosed by the gripper, a global coordinate system ($o_{w}(x_{w},y_{w},z_{w})$) is defined, and the coordinate transformation between the base coordinate systems of the three soft actuators and the global coordinate system is developed. The three base coordinate systems are defined as $o_{10}(x_{10},y_{10},z_{10})$, $o_{20}(x_{20},y_{20},z_{20})$, and $o_{30}(x_{30},y_{30},z_{30})$, respectively. The coordinate transformation between $o_{10}$ and $o_{w}$ can be given by

(29) $$T_{w1}=\left[\begin{array}{cc} R_{w1} & X_{w1}\\ 0 & 1 \end{array}\right]=\left[\begin{array}{cccc} \cos\alpha_{1} & -\sin\alpha_{1} & 0 & x_{w1}\\ \sin\alpha_{1} & \cos\alpha_{1} & 0 & y_{w1}\\ 0 & 0 & 1 & z_{w1}\\ 0 & 0 & 0 & 1 \end{array}\right]$$

where $X_{w1}={\left[\begin{array}{ccc} x_{w1} & y_{w1} & z_{w1} \end{array}\right]}^{T}$ is the vectorial coordinate of the base coordinate system $o_{10}$ relative to the global coordinate system. $R_{w1}$ is a rotation matrix from the base coordination to the global one, and it can be given by

(30) $$R_{w1}=\left[\begin{array}{ccc} \cos\alpha_{1} & -\sin\alpha_{1} & 0\\ \sin\alpha_{1} & \cos\alpha_{1} & 0\\ 0 & 0 & 1 \end{array}\right]$$

where $\alpha_{1}$ is the angle between the $x_{10}$- and $x_{w}$-axis. The coordinate transformation between the $o_{20}$ and $o_{w}$ can be given by

(31) $$T_{w2}=\left[\begin{array}{cc} R_{w2} & X_{w2}\\ 0 & 1 \end{array}\right]=\left[\begin{array}{cccc} \cos\beta_{1} & -\sin\beta_{1}\cos\beta_{2} & -\sin\beta_{1}\sin\beta_{2} & x_{w2}\\ \sin\beta_{1} & \cos\beta_{1}\cos\beta_{2} & \cos\beta_{1}\sin\beta_{2} & y_{w2}\\ 0 & -\sin\beta_{2} & \cos\beta_{2} & z_{w2}\\ 0 & 0 & 0 & 1 \end{array}\right]$$

where $X_{w2}={\left[\begin{array}{ccc} x_{w2} & y_{w2} & z_{w2} \end{array}\right]}^{T}$ is the vectorial coordinate of the base coordinate system $o_{20}$ relative to the global coordinate system, and the rotation matrix $R_{w2}$ can be given by

(32) $$R_{w2}=\left[\begin{array}{ccc} \cos\beta_{1} & -\sin\beta_{1}\cos\beta_{2} & -\sin\beta_{1}\sin\beta_{2}\\ \sin\beta_{1} & \cos\beta_{1}\cos\beta_{2} & \cos\beta_{1}\sin\beta_{2}\\ 0 & -\sin\beta_{2} & \cos\beta_{2} \end{array}\right]$$

where $\beta_{1}$ is the angle between the $x_{20}$- and $x_{w}$-axis, and $\beta_{2}$ is the angle formed by the $z_{20}$- and $z_{w}$-axis. Similarly, the transformation matrix between $o_{30}$ and $o_{w}$ can be given by

(33) $$T_{w3}=\left[\begin{array}{cc} R_{w3} & X_{w3}\\ 0 & 1 \end{array}\right]=\left[\begin{array}{cccc} \cos\gamma_{1} & -\sin\gamma_{1}\cos\gamma_{2} & \sin\gamma_{1}\sin\gamma_{2} & x_{w3}\\ \sin\gamma_{1} & \cos\gamma_{1}\cos\gamma_{2} & -\sin\gamma_{2}\cos\gamma_{1} & y_{w3}\\ 0 & \sin\gamma_{2} & \cos\gamma_{2} & z_{w3}\\ 0 & 0 & 0 & 1 \end{array}\right]$$

where $X_{w3}={\left[\begin{array}{ccc} x_{w3} & y_{w3} & z_{w3} \end{array}\right]}^{T}$ is the vectorial coordinate of the base coordinate system $o_{30}$ relative to $o_{w}$, and the rotation matrix $R_{w3}$ can be given by

(34) $$R_{w3}=\left[\begin{array}{ccc} \cos\gamma_{1} & -\sin\gamma_{1}\cos\gamma_{2} & \sin\gamma_{1}\sin\gamma_{2}\\ \sin\gamma_{1} & \cos\gamma_{1}\cos\gamma_{2} & -\sin\gamma_{2}\cos\gamma_{1}\\ 0 & \sin\gamma_{2} & \cos\gamma_{2} \end{array}\right]$$

where $\gamma_{1}$ is the angle between the $x_{30}$- and $x_{w}$-axis, and $\gamma_{2}$ is the angle formed by the $z_{30}$- and $z_{w}$-axis. Based on these coordinate transformation matrices, we can obtain the coordinates of the *j*th chamber joint of the *i*th soft actuator in the global coordinate system, and it can be written as

(35) $$P_{wji}=T_{wj}T_{bji}P_{ji}=T_{wj}T_{j1}T_{j2}\cdots T_{ji}P_{ji}$$

where $P_{ji}$ is the local coordinates of the *i*th chamber joint of the *j*th soft actuator, $T_{bji}$ is the coordinate transformation matrix from the local coordinate system to the base one, and $T_{wj}$ is the transformation matrix between the base coordinate system and the global one.

## 4. Validation

**Figure 8:**
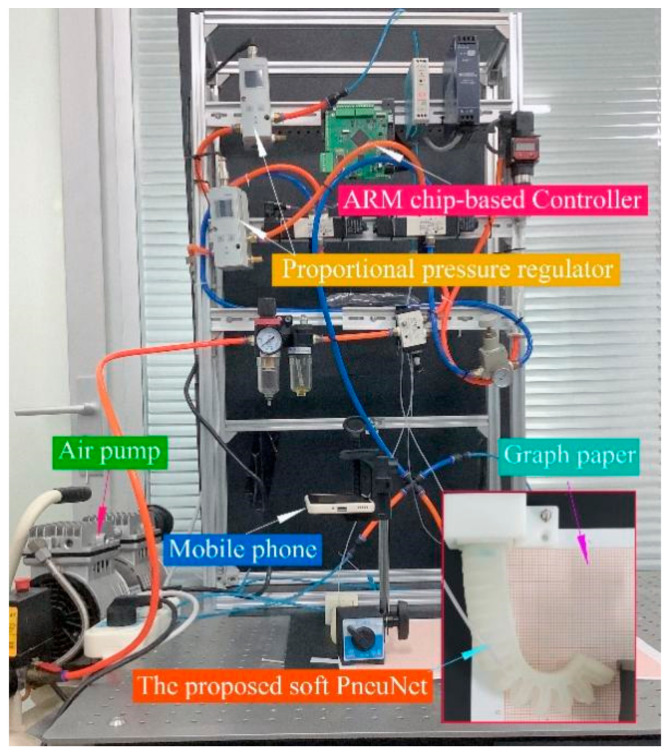
Experimental system.

To test the effectiveness of the models, an experimental system was built, as presented in Figure 8. Two proportional pressure regulators (VPPM-6L, Festo Ltd., Esslingen am Neckar, Germany), which have an output range of 0–600 kPa with a linearity error of ±1% FS, were used to adjust the input pressure of the actuators. A self-developed ARM microprocessor-based controller was used to control the outputs of the regulators. Module one of the actuator is controlled by one proportional pressure regulator, and module two by the other one. Since two modules are controlled by different regulators, the soft actuator can work at different pressure combinations. Moreover, graph papers with a minimum scale of one millimeter were utilized to offer information on the bending angle and location.

**Figure 9:**
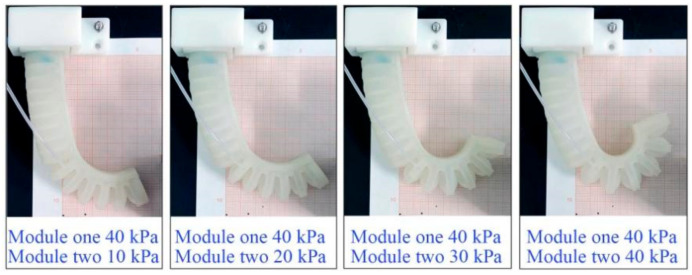
Deformation of the proposed actuator under different pressures.

During the trials, the pressure for module one of the soft actuator was limited to a range of 0–80 kPa with an increment of 10 kPa, and that for module two was limited to a range of 0–40 kPa with an interval of 5 kPa; thus, a total of 81 pressure combinations were used. Figure 9 shows the deformation of the actuator when the pressure of module one remains at 40 kPa and that of module two varies from 10 to 40 kPa. With the changes in pressure, the deformation of the soft actuator changes notably. For each combination, five inflating trials were performed. The vertical projection of the SPA on the graph paper was used to provide the bending angle of the soft actuator as well as the position data of the actuator’s tip in the base coordinate system. By utilizing a protractor and the scale on the graph paper, the bending angle and coordinate data can be read by visual measurement.

**Figure 10:**
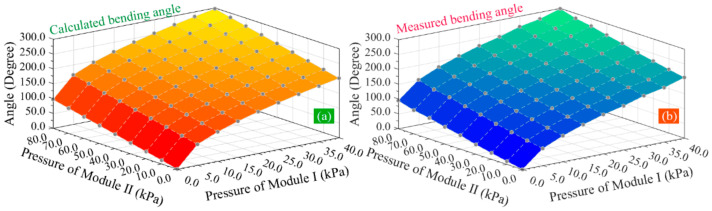
The bending angle of the soft actuator under different pressures: (**a**) the calculated bending angle and (**b**) the measured bending angle.

The average values of the measured results of five inflating trials were calculated. Figure 10a records the total bending angles predicted by the bending angle model, and Figure 10b shows the measured bending angles of the inflating experiments. It can be seen that the predicted and measured bending angles present a very similar tendency. With the increase in applied pressure for two pneumatic modules, the total bending angle of the SPA increases approximately linearly.

**Figure 11:**
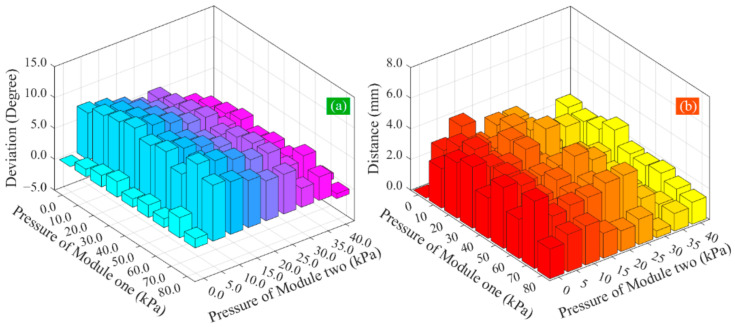
Comparison of the calculated and measured results: (**a**) bending angle and (**b**) location of the actuator’s tip.

Comparisons of the calculated and measured total bending angle are shown in Figure 11a. From the deviation between them, it can be seen that when the pressure of module two keeps constant, the deviation between the predicted and measured angle remains at a relatively stable state, indicating that the increase in the pressure of module one has little impact on the deviation. When the pressure of module one remains constant and that of module two is bigger than 5 kPa, the deviation tends to reduce with the increase in the pressure of module two, indicating that the pressure of module two has a more significant impact on the deviation, and the bending angle model can give a relatively accurate prediction when a bigger pressure is applied to module two of the soft actuator. Throughout the deviation, the maximum deviation is 9.13°, the minimum deviation is −2.28°, the mean deviation is 4.13°, and the standard deviation is 2.89°. When the pressure of module two is bigger than 5 kPa, all deviations are less than 8%. The comparison results show that the bending angle model has an expected agreement with the experimental results.

**Figure 12:**
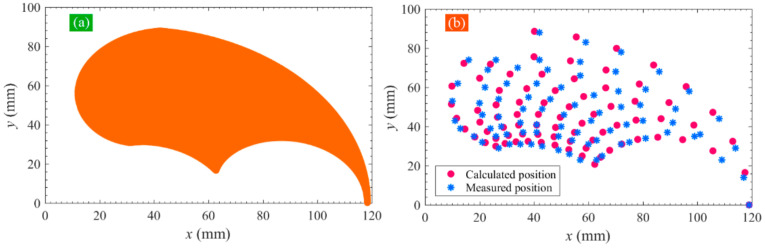
(**a**) Motion range of the proposed SPA predicted by the developed kinematic model and (**b**) comparison of the calculated and measured actuator’s tip location.

The motion of the soft actuator’s tip predicted by the kinematical model is shown in Figure 12a. For all pressure combinations mentioned above, the calculated tip locations are compared with the measured results in Figure 12b. It can be seen that the calculated and measured results present a nearly uniform distribution. To evaluate the position deviation, the distance between the predicted location and the corresponding measured location is calculated, and the results are shown in Figure 11b. It can be seen that the maximum distance is 4.05 mm, the minimum distance is 0.23 mm (except for the case where the input pressures of two modules are both zero), and the mean distance is 2.43 mm, showing a good prediction performance. The motion principle of the soft actuator can be explained well by the analytical kinematical model.

**Figure 13:**
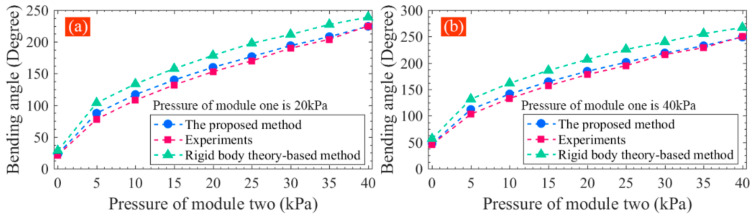
Comparison between the traditional and the proposed method for bending angle calculation: (**a**) pressure applied to pneumatic module one maintains at 20 kPa and (**b**) pressure applied to module one maintains at 40 kPa.

The bending angle model of the soft actuator is a basic model, and its performance determines the reliability of the kinematic model. As mentioned above, the conventional rigid-body-theory-based method is also widely used to model the bending angle, in which the soft actuator in action is viewed as a cantilevered beam. Based on the Euler–Bernoulli principle, the radius of curvature of the inflated actuator can be calculated, by which the bending angle can be obtained. A detailed introduction to this method can be found in [18]. To compare the performance of the traditional and proposed methods for the new soft actuator, the bending angle was also calculated using the conventional method. Here, two groups of comparisons are shown in Figure 13. In the first group comparison (Figure 13a), the pressure of pneumatic module one remains at 20 kPa, and that for module two changes from 0 to 40 kPa. In the other group comparison (Figure 13b), when the pressure of pneumatic module two varies from 0 to 40 kPa, that of module one stays unchanged at 40 kPa. It can be seen from Figure 13 that the bending angle calculated by the conventional method has bigger deviations from the practical measuring results. The proposed method presents better calculating precision, demonstrating that it has good performance.

**Figure 14:**
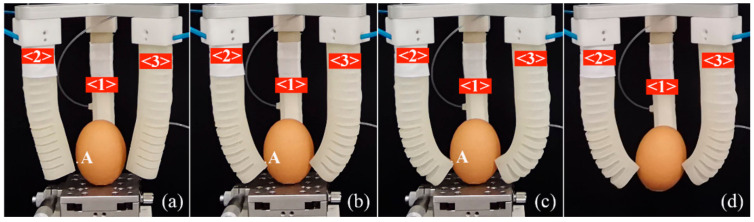
Grasping process of an egg: (**a**) the soft actuator approaches the egg when the pressure of pneumatic module one is 10 kPa and that of module two is zero; (**b**) the soft actuator’s tip can make contact with the egg when the pressure of module one is 20 kPa and that of module two is 14.1 kPa; (**c**) the gripper pinches the egg tightly when the pressure of module one is 20 kPa and that of module two is also 20 kPa; (**d**) the egg is grasped stably when the platform is removed.

Relying on the bending angle model and kinematic model, the grasping experiments of the three-finger soft gripper were carried out, and the pinching grasping and enveloping grasping were tested. First, an egg is selected as a target object. Before grasping, the egg is placed on a three-directional micro-positioning platform with a short piece of double-sided sticky tape. By adjusting the platform, the egg can be located in the middle of the space enclosed by the three soft actuators. Figure 14 records the primary stages of the grasping process. Take the second soft actuator in Figure 14a as an example. A target pinching position on the egg (point A) in the global coordinate system is (−7.5, 75, 12.9). Based on Equation (35), the coordinates of the pinching position can be transformed into the base coordinate system of the second soft actuator to obtain (75, 51.4, 0). Then, depending on the kinematic model (Equation (28)) of the soft actuator, the required bending angle can be calculated, and in this case, it is 100.4°. Thus, according to the bending angle models of Equations (12) and (20), an appropriate pressure combination can be determined, in which the pressure of module one is 20 kPa, and that of module two is 13.9 kPa.

When the pressure of pneumatic module one is set as 10 kPa, it can be seen from Figure 14a that the tips of the soft actuators edge a little close to the egg. At this time, a pressure of 14.1 kPa, which is slightly more than the theoretical value (13.9 kPa), is applied to pneumatic module two, and it can be seen the soft actuator’s tip just makes contact with the egg near the target point A, as shown in Figure 14b, showing that the developed bending angle and kinematic models work well. To realize the grasping, sufficient output force is needed; therefore, the pressure applied to module two increases to 20 kPa, making pneumatic module two generate a bigger bending deformation, as shown in Figure 14c. It should be noted that the bending angle model cannot be used during this stage since the reactive force of the egg on the soft actuator make the soft actuator less able to bend freely, destroying the original deformation and kinematic rules. Under this condition, an output force model is necessary, which can provide a suitable pressure combination to grasp the object stably. The output force model of the proposed SPA will be studied in future research. When we remove the platform, we can see that the egg can be grasped stably by the soft gripper, although the double-sided sticky tape tilts the egg at a small angle.

**Figure 15:**
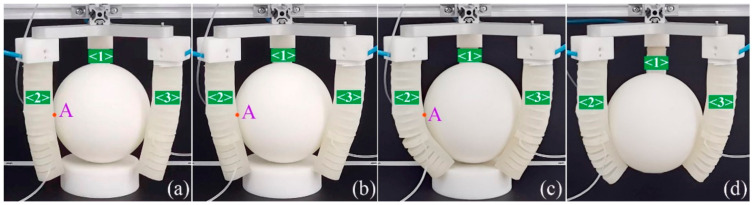
Grasping process of a sphere: (**a**) the soft actuator approaches the sphere when the pressure of module one is 25 kPa and that of module two is zero; (**b**) the soft actuator’s tip can contact the sphere when the pressure of module one is 29 kPa and that of module two is zero; (**c**) the gripper envelops the sphere tightly when the pressure of module one is 40 kPa and that of module two is zero; (**d**) the sphere is grasped stably when the heel block is removed.

To test the enveloping grasping, a sphere with a diameter of 100 mm was used, and its grasping process is shown in Figure 15. Take the second soft actuator as an example again. We hope that the seventh chamber of the pneumatic module one can get in touch with point A on the surface of the sphere, which has a coordinate of (−25, 55, 43.3) in the global coordinates. Depending on the matrix transformation and bending angle model of Equation (12), the required pressure was calculated, and it was 28.7 kPa. In Figure 15a, module one of the soft actuator is inflated by a pressure of 25 kPa, with the pressure of module two remaining at zero. It can be seen that the soft actuator approaches the sphere. When the pressure applied to module one increases to 29 kPa, the soft actuator can make contact with the target point, as Figure 15b shows. The used pressure is slightly greater than the theoretical value. To grasp the sphere, we continue to enhance the pressure of module one to 40 kPa, as shown in Figure 15c, and it can be seen that pneumatic module one can envelop the sphere. At this time, module two of the soft actuator is inflated by a pressure of 20 kPa, and the sphere is enveloped tightly by the soft actuator, forming a bigger contact area. When the heel block under the sphere is removed, as shown in Figure 15d, a stable enveloping grasping is realized. It should be noted, owing to the impact that the sphere’s shape has on the bending and kinematic behavior of the SPA, that the bending angle model and kinematic model will lose efficacy; instead, an output force model is required.

## 5. Conclusions

This investigation presents a new type of SPA for soft grippers used in grasping, which adopts a composite structure made of two kinds of pneumatic networks. Compared with the traditional single-pneumatic-network-based soft actuator, the new SPA combines the advantages of the two kinds of pneumatic network structures, presenting high reliability and stability, whether in pinching or enveloping grasping. To promote the practical application of the proposed soft actuator, this investigation aims to develop its bending angle model and kinematic model.

Based on the analyses of the bending principle and kinematic characteristics, the Yeoh constitutive model, moment equilibrium, virtual work principle, PPC method, and coordinate transformation were combined to model the bending angle and motion rules of the proposed soft actuator. To test the effectiveness of the developed analytical models, an experimental system was built up, and a series of inflating trials on the proposed SPA were carried out. Comparison between the calculated and measured bending angles shows that the maximum, minimum, mean, and standard deviation are 9.13°, −2.28°, 4.13°, and 2.89°, respectively. When the pressure of module two is bigger than 5 kPa, all deviations are less than 8%, and the calculated bending angle presents an expected agreement with the experimental results. To verify the performance of the kinematic model, the motion of the actuator’s tip was observed, and the distance between the calculated and measured positions was calculated. The test results show that the maximum distance is 4.05 mm, the minimum distance is 0.23 mm, and the mean distance is 2.43 mm, demonstrating that the developed kinematic model has a good prediction performance.

To test the performance of the developed bending angle model and kinematic model in practical grasping, a soft gripper consisting of three proposed soft actuators was assembled, and the pinching and enveloping grasping were tested. The results further demonstrate that the developed bending angle model and kinematic model are effective, laying a good foundation for the practical application of the proposed soft actuator. During the grasping trials, it was also found that the developed bending angle and kinematic models are effective up to the phase when the soft actuator is in contact with the object. However, after the soft actuator contacts the target object, the two models will be no longer operative; this is closely related to the interaction between the actuator and the object, as well as the impact that the object’s shape has on the proposed SPA. Under this condition, to grasp the object successfully, an output force model is necessary, and this will be studied in future research.

## Figures and Tables

**Table 1:** Parameters of the proposed SPA (unit: mm).

Parameters	Values	Parameters	Values	Parameters	Values	Parameters	Values
$w_{1}$	18	$r_{2}$	10	$l_{1}$	9	$t_{3}$	3
$w_{2}$	20	$r_{3}$	8	$l_{2}$	2	$t_{4}$	1
$w_{3}$	16	$r_{4}$	10	$l_{3}$	5	$t_{5}$	2
$w_{4}$	20	$h_{1}$	10	$t_{1}$	2	$t_{6}$	2
$r_{1}$	9	$h_{2}$	10	$t_{2}$	2	$t_{7}$	1
